# Analysis of the Changes in Occlusal Plane Inclination in a Class II Deep Bite “Teen” Patient Treated with Clear Aligners: A Case Report

**DOI:** 10.3390/ijerph19020651

**Published:** 2022-01-06

**Authors:** Edoardo Staderini, Valentina Ventura, Simonetta Meuli, Liliana Ávila Maltagliati, Patrizia Gallenzi

**Affiliations:** 1 Postgraduate School of Orthodontics, Fondazione Policlinico Universitario Agostino Gemelli IRCCS, 00168 Rome, Italy; venturav571@gmail.com (V.V.); simonetta.meuli@fastwebnet.it (S.M.); patrizia.gallenzi@unicatt.it (P.G.); 2 Postgraduate School of Orthodontics, Università Cattolica del Sacro Cuore, 00168 Rome, Italy; 3 Postgraduate School of Orthodontics, Guarulhos University, Guarulhos 07023-070, Brazil; liliana.maltagliati@prof.ung.br

**Keywords:** clear aligner appliance, Angle Class, occlusal plane, elastics, growth and development

## Abstract

*Background*: Optimal management of hypodivergent growing patients demands a strict control of vertical dimension and to exploit the growth potential. If a deep bite malocclusion causes a traumatic contact between the upper and lower incisors and affects the facial appearance, an early interceptive treatment is recommended. The aim of this case report is to outline the clinical management of the occlusal plane of a growing Class II division 1 deep bite patient treated with aligners and Class II elastics. *Methods*: The treatment lasted 11 months and was divided into two phases. Treatment goals included improvement of the soft tissue profile and basal bone relationships through an increase in the mandibular third of the face and a sagittal advancement of the mandible. The correction of the curve of Spee involved intrusion of the mandibular incisors and extrusion of the mandibular premolars. *Results*: The cephalometric analysis at the end of the treatment displayed significant differences in the skeletal and occlusal pattern along with aesthetic improvements. *Conclusion*: The final cephalogram showed a consistency between the planned tooth movement and the clinical results. Although definitive recommendations must be withheld until longer follow-up is available, the patient presented here shows that the treatment protocol yielded positive mandibular growth.

## 1. Introduction

Vertical development is related to rotation of the maxillary bones.

Studies developed by Bjork et al. [1] highlighted that the growth of the mandible with respect to the cranial basis is characterized by two processes: internal and external rotation. Internal rotation is composed of two processes: rotation around the condyle (25%) and rotation around the center of the mandible (75%). The external rotation is due to the remodeling processes on the anteroinferior (apposition) and posteroinferior (resorption) border of the mandible.

The superimposition of lateral cephalometric film based on implants showed that mandibular rotation during eurythmic craniofacial growth has a little influence on mandibular plane inclination since a forward internal rotation of 15°, on average, is compensated by an external backward rotation of about 11°–12°, thus determining a reduction in the mandibular plane angle of 3°–4°, approximately.

Concerning the maxilla, it is possible to observe a forward internal rotation, even if a backward rotation frequently occurs. There is an external rotation because of apposition on the palatal side and resorption on the nasal cavity, and because of the eruption of incisors and molars. In most cases, the internal and external rotation counteract each other and do not modify the inclination of the palatal plane. Short face patients show forward rotation of the mandible due to an increased internal rotation and a reduced external rotation: they are usually characterized by an almost horizontal palatal plane (parallel to the true horizontal line) and an occlusal plane with the presence of a deep bite.

Long face patients show backward rotation of the mandible due to an absent or reversed internal rotation of the mandible, mostly due to rotation around the condyle rather than the center of the mandible: they are characterized by a backward rotation of the palatal plane and occlusal plane, with the presence of an open bite.

Studies developed by Sato et al. [2,3], highlighted the correlation between maxillofacial growth and occlusal plane (OP) inclination: the parallel inclination (flatness) or backward rotation (steepness) of the palatal plane are due to rotation in flexion and extension of the sphenoid–occipital complex, respectively. According to Sato’s theory, the occlusal plane inclination is established by the occlusion. In a growing facial skeleton, the position of the OP is determined largely by the vertical growth of the maxillary teeth, and the inclination of the OP is determined largely by the growth of the dentoalveolar bone. 

The current trend for clear aligners’ treatment is to address growing patients, but there is poor literature about the use of clear aligners and class II elastics to promote mandibular sagittal and vertical growth: in fact, the thickness of the aligners itself may be used as a tool for achieving a proper control of posterior vertical dimension. The aim of this case report is to provide the management of the occlusal plane of a growing hypodivergent Class II division 1 patient treated with clear aligners in combination with Class II elastics. This study was reported according to the CARE checklist (Supplementary Materials Table S1).

## 2. Materials and Methods

### 2.1. Diagnosis

**Figure 1:**
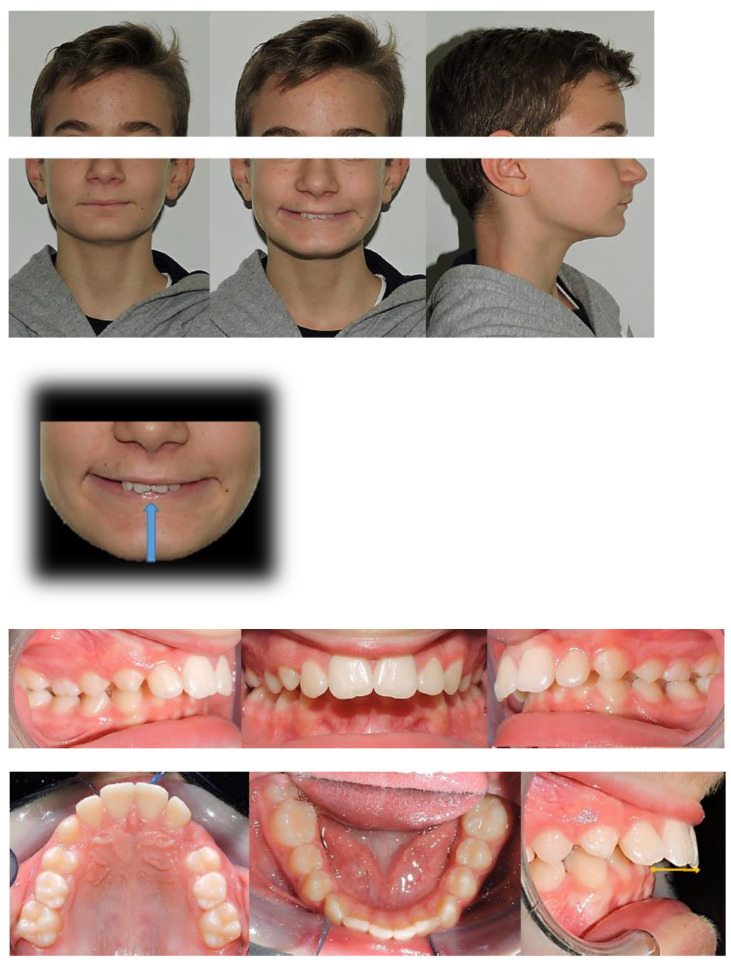

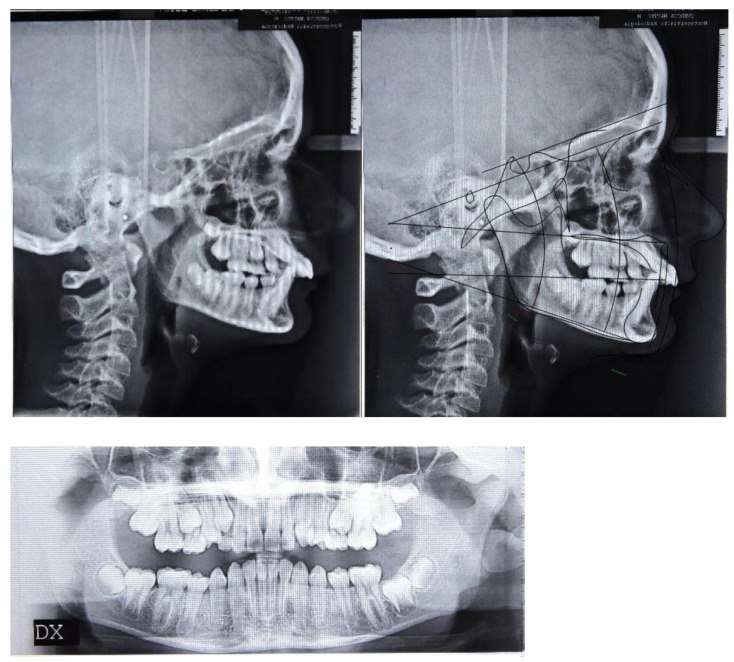
Pre-treatment intraoral, extraoral photographs and radiographic examination.

The patient was a 12.7-year-old Caucasian male with a chief complaint of excessive exposure of upper incisors (Figure 1). The general medical history was negative for illness, allergy. The patient did not present any familiarity for Angle Class II malocclusion and had not received any previous orthodontic treatment. Facial photographs revealed a convex profile with a hypodivergent growth pattern. At intraoral evaluation, the patient showed palatal impingement of the mandibular incisors on the palatal mucosa, as well as mandibular lip interposition between upper and mandibular incisors; narrow upper and mandibular arches (maxillary intermolar width-distance between maxillary first molar palatal cusp tips: 43 mm; mandibular intermolar width-distance between the central fossa of mandibular first molars: 46 mm), a bilateral molar and canine Angle Class II relationship, increased overjet and overbite with a deep curve of Spee were observed (Figure 1). 

In accordance with the Radiographic Guidelines of the British Orthodontic Society (last access: 1 March 2021, https://www.bos.org.uk), lateral cephalometric radiographs were obtained and analyzed. Landmarks and measurements were validated by Shaw et al. [4], and all data were anonymized.

The orthopantomography revealed a late mixed dentition, with the absence of “tooth developmental anomalies” of number, size, shape, eruption, position, structure.

According to Steiner’s cephalometric analysis [5], the patient showed a skeletal Class II relationship (ANB = 6°; Wits = 4 mm). Referring to the anterior cranial base (SN plane: Sella-Nasion), the patient presented a retruded mandible (SNB = 75°) with an increased inclination of upper incisors (U1-SN = 125°; IMPA = 90°) (Figure 1). The overjet and the overbite were also increased at the beginning of treatment (overjet = 8 mm; overbite = 6 mm) (Figure 1).

According to the Sassuoni analysis of vertical facial proportions [6], the patient revealed a skeletal deep bite tendency as the anterior cranial base plane, the Frankfort plane (FH), the palatal plane and the mandibular plane tended to converge far behind the face. However, the mandibular plane and the occlusal plane intersected relatively close to the face, owing to a deep curve of Spee with extrusion of mandibular incisors. (Figure 1). According to Steiner’s cephalometric analysis, it was confirmed that the occlusal plane (OP) was almost parallel to Frankfort’s plane (OP-FH = 2°), and that the maxilla (palatal plane, ANS/PNS) was properly inclined with respect to Frankfort’s plane (ANS/PNS-FH = 4°); moreover, the angle (FMA) between mandibular plane (ML) and Frankfort’s plane (FH) revealed a forward rotation of the mandible at the beginning of the treatment (FMA = 19°). 

According to the cervical vertebral maturation method [7], the patient was in a pubertal phase without completing his craniofacial growth (between CS2 and CS3) (Figure 1). 

### 2.2. Treatment Goals

Treatment goals included to improve soft tissue profile and basal bone relationship, through an increase in the mandibular third of the face and a sagittal advancement of the mandible. The correction of deep bite and curve of Spee involved the management of the occlusal plane through intrusion of mandibular incisors and extrusion of premolars.

### 2.3. Treatment Alternatives

The first treatment option was a functional appliance treatment. However, this hypothesis was rejected as it provides a relative intrusion of mandibular incisors. The second treatment option was a fixed appliance treatment; however, the patient was still in a late mixed dentition. Therefore, starting before completion of permanent dentition would lead to increase in the duration of treatment, whereas if the fixed treatment was delayed after the completion of the permanent dentition, there would be a risk to the mandibular response because the patient could be far from growth spurt. The final treatment option was clear aligners combined with Class II elastics; the Class II elastics can be used as functional devices in late mixed dentition promoting the sagittal growth of the mandible [5].

### 2.4. Treatment Plan

**Figure 2:**
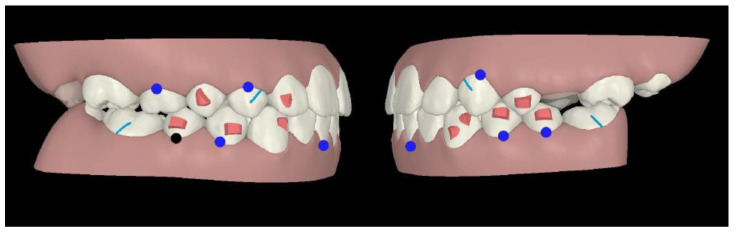
Treatment plan phase I.

An Invisalign^®^ “Teen” package was chosen, as it allowed to ask for a multi-phase treatment: in fact, it is possible to ask for additional aligners at any stage of tooth eruption. Compliance indicators were provided on the buccal area of the aligners in order to monitor patient’s adherence during treatment. The first phase of treatment was scheduled in late mixed dentition: horizontal attachments were placed over mandibular first and second bicuspids as posterior anchorage to allow intrusion forces of mandibular incisors area, whereas incisors did not require attachments (Figure 2).

On the maxillary right first bicuspid, the optimized rotation attachment was placed for its distal rotation and lingual inclination (Figure 2).

On the maxillary right lateral incisor, an optimized attachment on the labial surface and a pressure point on lingual surface were placed to achieve intrusion and mesial rotation (Figure 2).

On the mandibular left canine, an optimized attachment was planned for root control during its mesial rotation and distal translation, whereas on the mandibular right canine the optimized attachment was placed for its mesial rotation. Moreover, their intrusion was planned (Figure 2).

**Figure 3:**
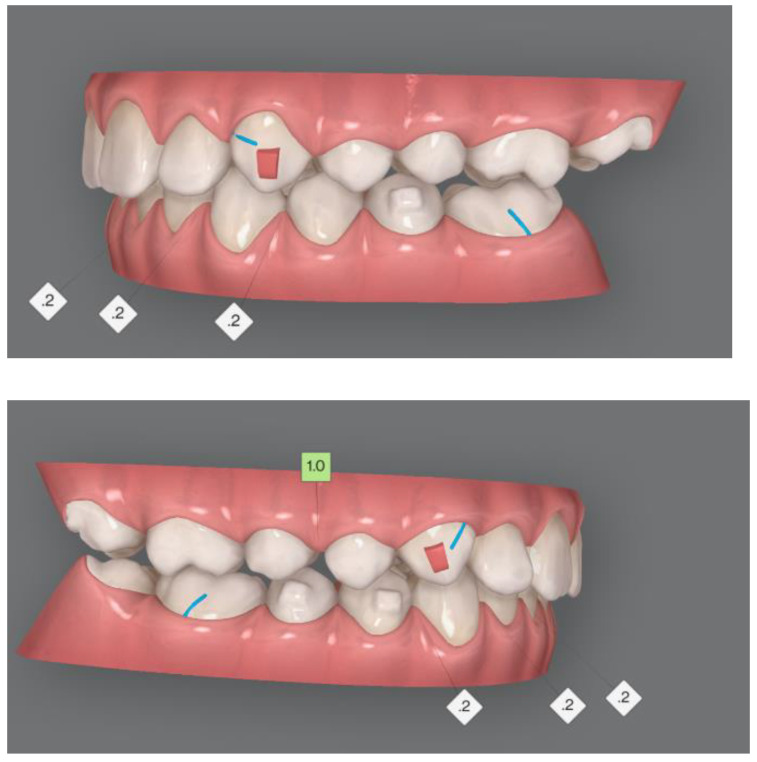
Treatment plan phase II.

In first and second phase, precision cuts were designed on the aligner surface for Class II elastics in order to obtain a mandibular advancement (Figure 2 and Figure 3).

The second phase of treatment was scheduled in complete permanent dentition. Rectangular vertical attachments were placed on the maxillary canines for root control and retention for interarch elastics, whereas horizontal attachments over mandibular right bicuspids and second left bicuspid provided anchorage for intrusion of mandibular incisors (Figure 3).

### 2.5. Treatment Progress

Upper and mandibular arch impression and bite registration were taken with iTero Element intraoral scanner (3 Shape Dental System, Copenhagen, Denmark) and set to Align Technology^®^. 

A three-dimensional virtual planning of tooth movement was performed through ClinCheck^®^ software version 5.6, Align Technology, San Jose, CA, USA). Written informed consent was obtained from the patient for the treatment plan, the publication of this short report and any accompanying images.

The treatment started when the patient was 12.7 years old. The first phase involved 20 aligners, and a 10-days aligner change protocol was adopted. The patient was instructed to wear aligners at least 20/22 h a day, except for meals and brushing. The patient was motivated to maintain good oral hygiene.

To provide retention for interarch elastic use, precision cuts were designed on the aligner surface of maxillary canines and mandibular first molars. Class II elastics (3M Unitek) were 8 h/day, with 4.5 oz. force and 3/16-inch lumen size.

In the second phase, an interproximal reduction of 2 mm was performed on the anterior mandibular arch from first right bicuspid to second left bicuspid (Figure 3). 

The first phase finished after 6 months of therapy. The second phase started when the patient was 13.3 years old and lasted 4.9 months. Twenty-one aligners were scheduled, and a 7-day aligner change protocol was adopted. 

In the second phase, the patient wore Class II elastics, with the abovementioned protocol.

## 3. Results

The overall treatment lasted 11 months and included 41 aligners. The use of compliance indicators provided an optimal patient’s adherence to treatment, without any adverse effects reported.

**Figure 4:**
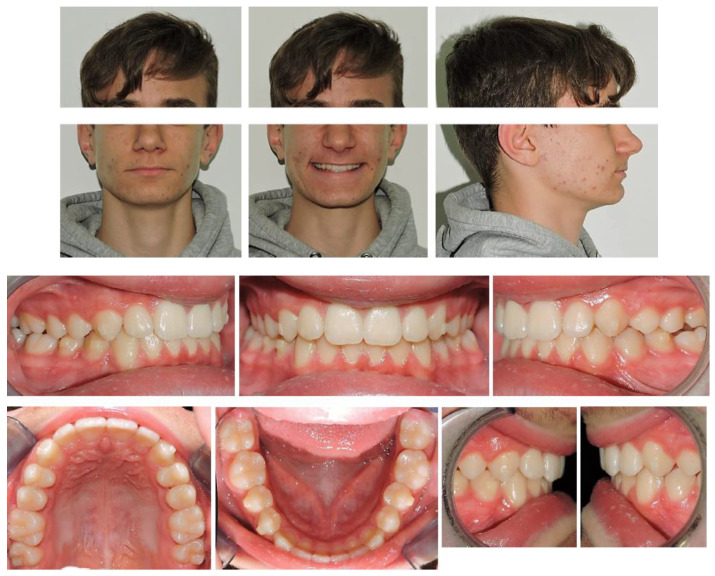

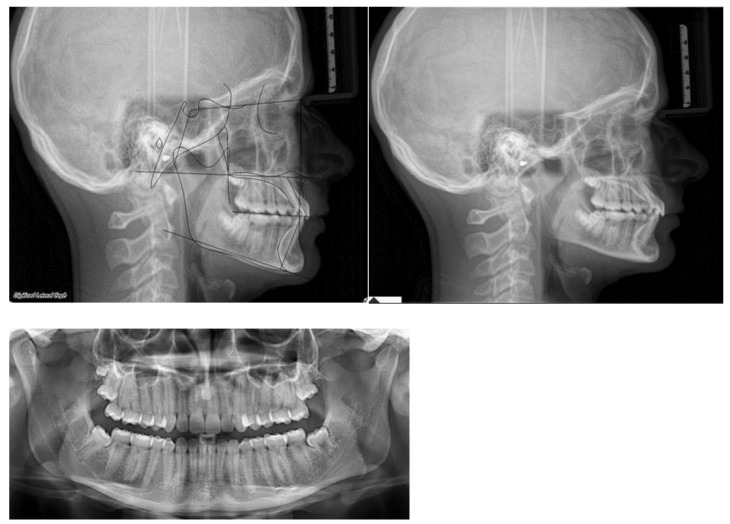
Post-treatment intraoral, extraoral photographs and radiographic examination.

At clinical examination, dental Class I and crowding correction, with proper incisor inclination, and ideal overjet and overbite were achieved (Figure 4).

Follow-up orthopantomography showed good root parallelism, without any sign of crestal bone loss and apical root resorption; however, a supernumerary maxillary right tooth was noticed (Figure 4). 

Post-treatment lateral teleradiograph showed skeletal, dental and aesthetic improvements (Table 1 and Figure 4)

**Figure 5:**
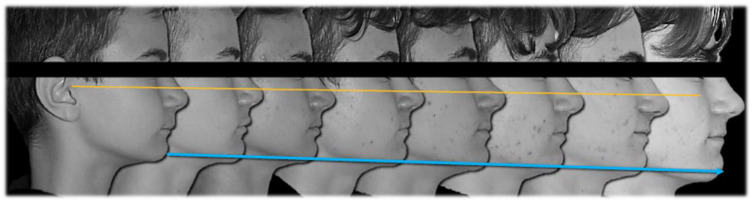
Aesthetic changes.

- Skeletal outcomes: cephalometric analysis according to Bjork showed a skeletal Class I relationship (ANB = 2°, Wits = 3 mm). Referring to the anterior cranial base (SN plane: Sella-Nasion), the patient presented a properly sagittal (SNB = 81°) and vertical position of the mandible with an increase in the angle (FMA) between the mandibular plane (ML) and Frankfort’s plane (FH) at the end of treatment (FMA = 21°)
- Dental outcomes: the cephalometric analysis according to Down revealed a change in the inclination of the occlusal plane referring to Frankfort’s plane (OP-FH = 5°), whereas the angle between the maxilla (palatal plane, ANS/PNS) and the Frankfort’s plane was unchanged at the end of treatment (ANS/PNS-FH = 4°). The inclination of the upper incisors was reduced at the end of treatment (Sup/SN = 112°) along with a flat curve of Spee and proper overjet (OJ = 5 mm) and overbite (OB = 4 mm). The inclination of the mandibular incisors remained the same at the end of treatment (IMPA = 90°).
- Aesthetic outcomes: the soft tissue changes involved a straight profile with the jaws proportionately positioned in the sagittal plane. On a frontal view the patient showed an increase in anterior vertical dimension along with an ideal smile arc. From a functional point of view, the lip interposition between the upper and mandibular incisors was corrected (Figure 4 and Figure 5).

## 4. Discussion

**Figure 6:**
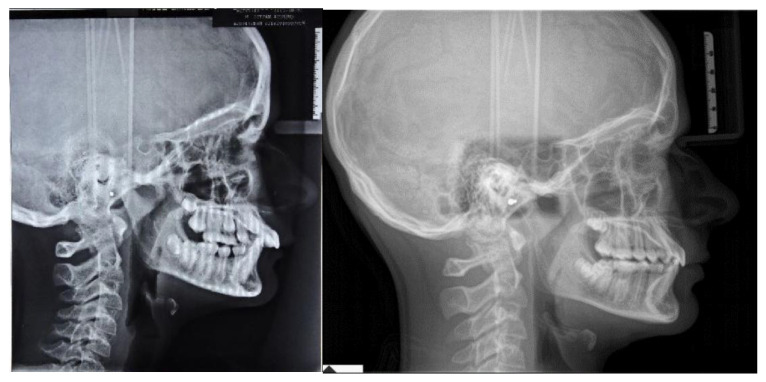
Cephalometric changes.

Post-treatment lateral teleradiograph showed a significant improvement in the sagittal bone relationship (Table 1), along with good vertical control, and a correct inclination of the upper and mandibular incisors. (Figure 4). A good proportion between the upper and mandibular arch width and shape was achieved along with a flat curve of Spee. Functional and aesthetic outcomes were stable at one-year follow-up and four-year follow-up (Figure 4, Figure 5 and Figure 6).

Many authors have reported that the three principal factors that seem to determine dental occlusal relationships are (1) potential differential maxillary and mandibular skeletal growth expressed along the occlusal plane; (2) the natural change in the cant of the occlusal plane during growth and development; (3) the Leeway space [5]. Occlusal plane inclination undergoes an anterior rotation during growth, both with reference to the cranial base, Frankfort plane and palatal plane [8]. Each degree of rotation of the occlusal plane will result in a half millimeter change in the dental occlusal relationship [7]. 

Many authors have stated that Class II facial types are characterized by backward rotation of the occlusal plane [8]. In short face patients, the steepness of the occlusal plane is associated with a backward rotation of the mandible; in a growing facial skeleton, it is therefore important to control the vertical growth of the maxillary teeth [2]. In fact, it has been stated that in Angle Class II malocclusion, those patients exhibiting the greatest growth during treatment exhibited the least change in the inclination of the occlusal plane; conversely, those cases exhibiting the least growth during treatment exhibited the greatest change in the occlusal plane [9]. 

Moreover, the Class II elastics induce extrusion of the mandibular molars and maxillary incisors, thus worsening the downward and backward inclination of the occlusal plane [8]. 

This study showed that clear aligners may address the unfavorable side effects of Class II elastics on vertical growth through modifying the inclination of the occlusal plane [5]; moreover, clear aligners can counter the side effects of Class II elastics on the proclination of the lower incisors [10]. As a matter of fact, at the end of treatment the inclination of the mandibular incisors remained unchanged. Clear aligners can be effective for intruding the mandibular anterior teeth. With a computer-aided pre-treatment tooth movement plan, the space required for intrusion can be accurately designed and predicted. In addition, aligners cover the entire dentition as an overlay appliance, thereby preventing extrusion of the posterior teeth.

However, the main limitation of the present study is the limited sample: further studies are required to investigate the effectiveness of the aligner treatment on the vertical control of the occlusal plane in growing patients. Moreover, dental patient-reported outcome measures of perceived oral health (oral function, orofacial pain, facial appearance, psychosocial impact) was not reported; even if a survey can allow researchers to collect some relevant data for clinical practice, the reliability of self-reported questionnaires for children is still controversial. [11,12].

## 5. Conclusions

This study reported the results of a comprehensive treatment with clear aligners of a Class II division 1 malocclusion in a “teen” patient. Although definitive recommendations must be withheld until longer follow-up is available, the combination between clear aligners and Class II elastics may help to address sagittal growth development together with a good vertical control of occlusal plane inclination [13,14,15].

## Figures and Tables

**Table 1:** Cephalometric values.

Values	Pre-Treatment	Post-Treatment
SNA (°)	81	83
SNB (°)	75	81
ANB (°)	6	2
FMA (°)	19	21
ANS/PNS-FH (°)	4	4
OP-FH (°)	2	5
U1-SN (°)	125	112
IMPA (°)	90	90
Wits (mm)	4	3
Overjet (mm)	8	5
Overbite (mm)	6	4

SNA: Sella-Nasion-Subspinale Angle (S: Sella; N: Nasion; A: subspinale point). SNB: Sella-Nasion-Supramentale Angle (S: Sella; N: Nasion; B: supramentale point). ANB: Subspinale- Nasion-Supramentale Angle. FMA: Frankfort Mandibular Plane Angle. U1-SN: Upper Incisor to Sella-Nasion Angle. IMPA: Incisor Mandibular Plane Angle. Wits: Wits Appraisal. Overjet: horizontal overlap of the incisors. Overbite: vertical overlap of the incisors. OP-FH: Occlusal Plane–Frankfort Plane Angle (OP: occlusal plane; FH: plane from Porion to Orion). PP-FH: Palatal plane–Frankfort Plane Angle (PP: plane from anterior nasal spine to posterior nasal spine).

## Data Availability

There are no linked research data sets for this submission. Data will be made available on request.

## Supplementary Materials

The following supporting information can be downloaded at: https://www.mdpi.com/article/10.3390/ijerph19020651/s1, Table S1: CARE checklist.
