# Supplementary material for: Analysis of the Changes in Occlusal Plane Inclination in a Class II Deep Bite “Teen” Patient Treated with Clear Aligners: A Case Report
Source: Int J Environ Res Public Health. 2022 Jan 6;19(2):651. doi: 10.3390/ijerph19020651 (PMC8775430; doi:10.3390/ijerph19020651)
Supplement: Supplementary file 1 [file ijerph-19-00651-s001.zip › ijerph-1461851-supplementary.pdf]

CARE case report guidelines

CARE Checklist of information to include when writing a case report

Topic	Item	Checklist item description	Reported on Line
**Title**	1	The diagnosis or intervention of primary focus followed by the words "case report'	1
Key Words	2	2 to 5 key words that identify diagnoses or interventions in this case report, including "case report"	1
Abstract (no references)	3a	Introduction: What is unique about this case and what does it add to the scientific literature?	1
	3b	Main symptoms and/or important clinical findings	1
	3c	The main diagnoses, therapeutic interventions, and outcomes	1
	3d	Conclusion—What is the main 'take-away" lesson(s) from this case?	1
Introduction	4	One or two paragraphs summarizing why this case is unique (may include references)	2
Patient Information	5a	De-identified patient specific information.	2
	5b	Primary concerns and symptoms of the patient.	2
	5c	Medical, family, and psycho-social history including relevant genetic information	2
	5d	Relevant past interventions with outcomes	N/A
Clinical Findings	6	Describe significant physical examination (PE) and important clinical findings.	3
Timeline	7	Historical and current information from this episode of care organized as a timeline	N/A
Diagnostic Assessment	8a	Diagnostic testing (such as PE, laboratory testing, imaging, surveys)	3
	8b	Diagnostic challenges (such as access to testing, financial, or cultural)	N/A
	8c	Diagnosis (including other diagnoses considered)	3
	8d	Prognosis (such as staging in oncology) where applicable	N/A
Therapeutic Intervention	9a	Types of therapeutic intervention (such as pharmacologic, surgical, preventive, self-care)	4, 5
	9b	Administration of therapeutic intervention (such as dosage, strength, duration)	5
	9c	Changes in therapeutic intervention (with rationale)	4
Follow-up and Outcomes	10a	Clinician and patient-assessed outcomes (if available)	6
	10b	Important follow-up diagnostic and other test results	6
	10c	Intervention adherence and tolerability (How was this assessed?)	6
	10d	Adverse and unanticipated events	N/A
Discussion	11a	A scientific discussion of the strengths AND limitations associated with this case report	9
	11b	Discussion of the relevant medical literature with references.	9
	11c	The scientific rationale for any conclusions (including assessment of possible causes)	10
	11d	The primary 'take-away" lessons of this case report (without references) in a one paragraph conclusion	9

Patient Perspective	The patient should share their perspective in one to two paragraphs on the treatment(s) they received	N/A
	Did the patient give informed consent? Please provide if requested………5……………………………………………	Yes No

Patient Perspective 12

Informed Consent 13
